# Sulforaphane inhibits growth and blocks Wnt/β-catenin signaling of colorectal cancer cells

**DOI:** 10.18632/oncotarget.26125

**Published:** 2018-09-21

**Authors:** Dominic B. Bernkopf, Gabriele Daum, Martina Brückner, Jürgen Behrens

**Affiliations:** ^1^ Experimental Medicine II, Nikolaus-Fiebiger-Center, Friedrich-Alexander University Erlangen-Nürnberg (FAU), 91054 Erlangen, Germany

**Keywords:** colorectal cancer, β-catenin, sulforaphane, Wnt signaling, TCF

## Abstract

The naturally occurring isothiocyanate sulforaphane (SFN) from cruciferous vegetables is associated with growth inhibition of various cancer types, including colorectal cancer. Colorectal cancer is most frequently driven by hyperactive Wnt/β-catenin signaling. Here, we show that SFN treatment reduced growth of three unrelated colorectal cancer cell lines (SW480, DLD1 and HCT116) via induction of cell death and inhibition of proliferation. Importantly, SFN inhibits Wnt/β-catenin signaling in colorectal cancer cells as shown by inhibition of β-catenin-dependent luciferase reporters and repression of β-catenin target genes (*AXIN2*, *LGR5*). SFN inhibits Wnt signaling downstream of β-catenin degradation and induces the formation of nuclear β-catenin structures associated with closed chromatin. Co-expression of the transcription factors LEF1 or TCF4 prevented formation of these structures and rescued inhibition of Wnt/β-catenin signaling by SFN. Our findings provide a molecular basis explaining SFN effects in colorectal cancer cells and underline its potential for prevention and therapy of colorectal cancer.

## INTRODUCTION

Colorectal cancer is one of the leading causes for cancer-associated morbidity and mortality in industrialized countries therefore representing a major health issue [1]. The vast majority of colorectal carcinomas are initiated by mutations which activate the Wnt/β-catenin signaling pathway [2].

The Wnt/β-catenin signaling pathway is an evolutionary conserved pathway involved in regulating embryonic patterning of body axes, stem cell fate and tissue homeostasis [3]. A destruction complex consisting of the tumor suppressor adenomatous polyposis coli (APC), axin, casein kinase 1α (CK1α) and glycogen synthase kinase 3 (GSK3) tightly regulates β-catenin abundance by inducing phosphorylation of β-catenin thereby triggering its ubiquitination and proteasomal degradation [4]. Binding of Wnt ligands to pairs of frizzled receptors and low-density lipoprotein receptor-related 5/6 (Lrp5/6) co-receptors leads to dishevelled (Dvl)-mediated inhibition of β-catenin phosphorylation and consequent stabilization of β-catenin [5]. In the nucleus, stabilized β-catenin interacts with T-cell factor (TCF)/lymphocyte enhancer factor (LEF) transcription factors to induce transcription of its target genes, e.g. *LGR5*, *AXIN2*, *MYC* and *CCND1* [6–10].

In more than 90% of all colorectal carcinomas, degradation of β-catenin is impaired by e.g. truncating loss of function mutations of APC or stabilizing gain of function mutations of β-catenin resulting in constant β-catenin accumulation and uncontrolled Wnt/β-catenin signaling activity [2]. Mitogenic β-catenin target genes like *MYC* and *CCND1* initiate cell division and fuel cancer growth.

Sulforaphane (SFN) is a naturally occurring isothiocyanate which is found in cruciferous vegetables such as broccoli [11]. Evidence is growing that SFN can inhibit growth of various cancer types derived from different organs thereby arousing interest to use SFN in anti-cancer therapy [12–14]. Consequently, SFN was used in a phase II study in men with recurrent prostate cancer and effort is made to optimize SFN production or to develop novel phosphonate analogs [15–17].

Some studies also showed inhibition of colorectal cancer growth by SFN [18, 19]. However, no common molecular mechanism has been revealed to explain SFN function in colorectal cancer cells. Of note, inhibition of colorectal cancer growth by SFN has not been linked to inhibition of Wnt/β-catenin signaling yet, although hyperactive Wnt/β-catenin signaling is the major driving force of colorectal cancer.

Here, we show SFN-induced growth inhibition of colorectal cancer cells and reveal that SFN is a potent inhibitor of Wnt/β-catenin signaling in colorectal cancer cells. Inhibition of Wnt/β-catenin signaling by SFN occurred downstream of β-catenin degradation, most likely at the level of β-catenin-TCF transcription complex formation, explaining why SFN is still active in mutated colorectal cancer cells.

## RESULTS

### SFN inhibits growth of colorectal cancer cells

**Figure 1:**
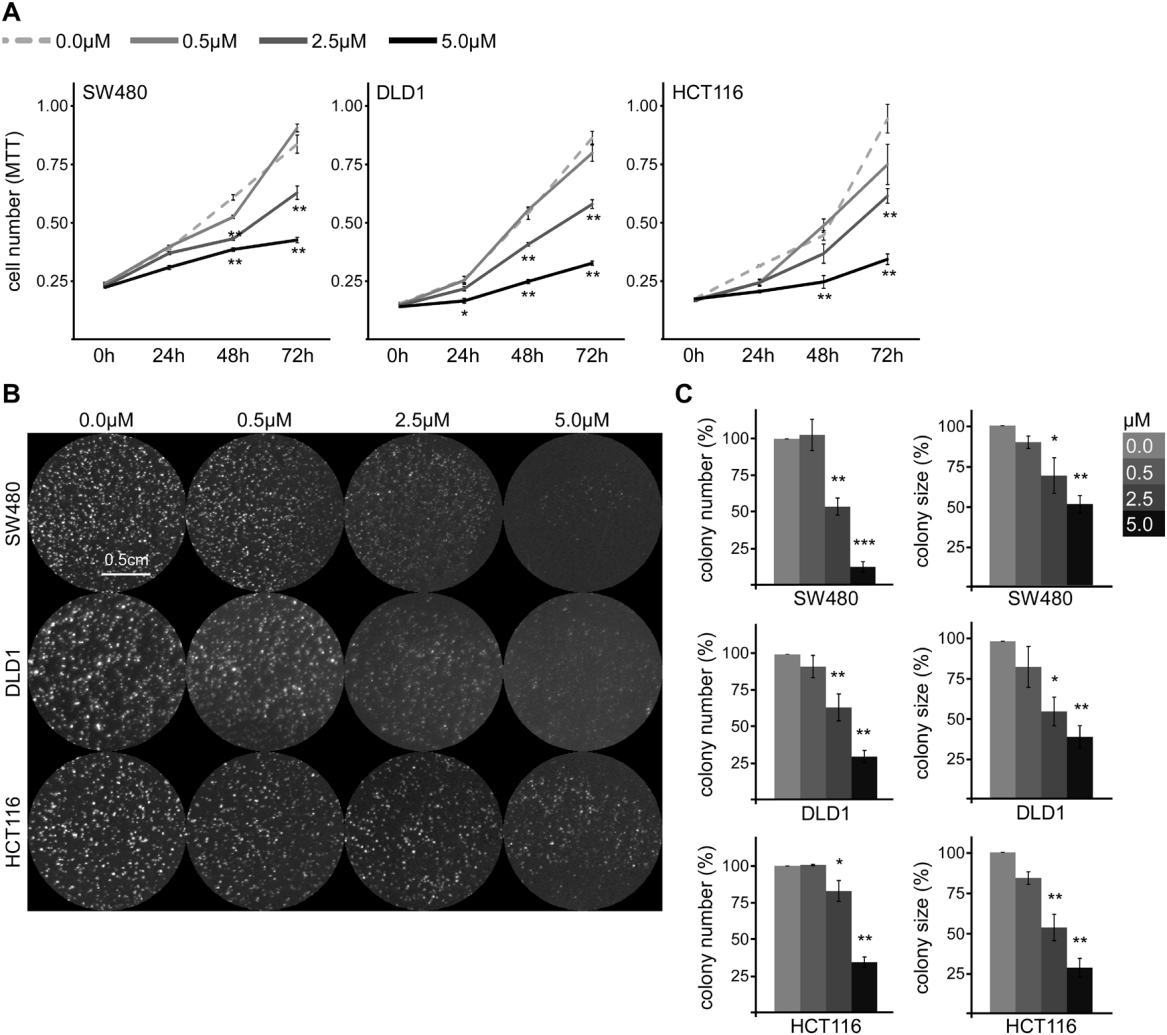
SFN inhibits growth of colorectal cancer cells **(A)** Violet MTT color intensity reflecting the number of viable SW480 (left panel), DLD1 (middle panel) or HCT116 cells (right panel) one day after seeding (0 h) or after 24 h, 48 h and 72 h of treatment with indicated SFN concentrations. One out of three representative experiments is shown. Results are mean +/− SEM of four replicates (n=4). ^*^p<0.05, ^**^p<0.01 (ANOVA followed by post hoc Tuckey test). **(B)** Cell colonies grown for 96 h from individual SW480, DLD1, or HCT116 cells in the presence of indicated SFN concentrations. Cells were stained by ethidium bromide incorporation and visualized with UV light. **(C)** Automated quantification of colony numbers (left column) and sizes (right column) from four independent experiments as in B. Results are mean +/− SEM (n=4). ^*^p<0.05, ^**^p<0.01, ^***^p<0.001 (Student's *t* test).

In this study we want to address whether SFN might inhibit growth of colorectal cancer by inhibiting Wnt/β-catenin signaling. As a model system we used two unrelated colorectal cancer cell lines with truncating APC mutations (SW480, DLD1) and one with a stabilizing β-catenin mutation (HCT116). To determine the effect of SFN on cell growth, SW480, DLD1 and HCT116 cells were treated with different concentrations of SFN (0, 0.5, 2.5 and 5 μM) for 24, 48 or 72 h within their logarithmic proliferation phase. Afterwards, the number of viable cells was assessed by colorimetric measuring of 3-(4,5-dimethylthiazol-2-yl)-2,5-diphenyltetrazolium bromide (MTT) reduction. Of note, SFN significantly inhibited cell growth in a dose-dependent manner in all three cell lines, with an IC50 of 3.7 μM for SW480, 3.5 μM for DLD1 and 3.6 μM for HCT116 cells (Figure 1A). After 72 h of 5 μM SFN treatment cell numbers of SW480, DLD1 and HCT116 cells were reduced by about 67, 73 and 78%, respectively, as compared to growth of untreated controls (Figure 1A). To validate the MTT assay-based results, we performed colony formation assays. In addition to cell growth, this assay measures the ability of single cells to grow out into colonies, a process required for metastasis formation. Treatment of cells with SFN during colony formation significantly reduced the numbers and sizes of colonies for the cancer cell lines SW480, DLD1 and HCT116 in a dose-dependent manner (Figure 1B, 1C). Moreover, SFN treatment inhibited colony formation of three additional colorectal cancer cell lines (CX-1, SW48 and WiDr) indicating broad responsiveness of colorectal cancer cells to SFN (Supplementary Figure 1). Interestingly, in contrast to colorectal cancer cells which depend on Wnt/β-catenin signaling to grow, colony formation of U2OS cells, whose growth is independent of Wnt signaling, was significantly less impaired (Supplementary Figure 1).

Together our experiments show that SFN inhibits growth of colorectal cancer cells. Interestingly, SFN was active at concentrations similar to those achieved by oral SFN uptake in a clinical study [15].

### SFN induces cell death and inhibits proliferation of colorectal cancer cells

Next, we wanted to determine whether reduced cell numbers after SFN treatment were due to induction of cell death by SFN and/or due to SFN-induced inhibition of proliferation.

**Figure 2:**
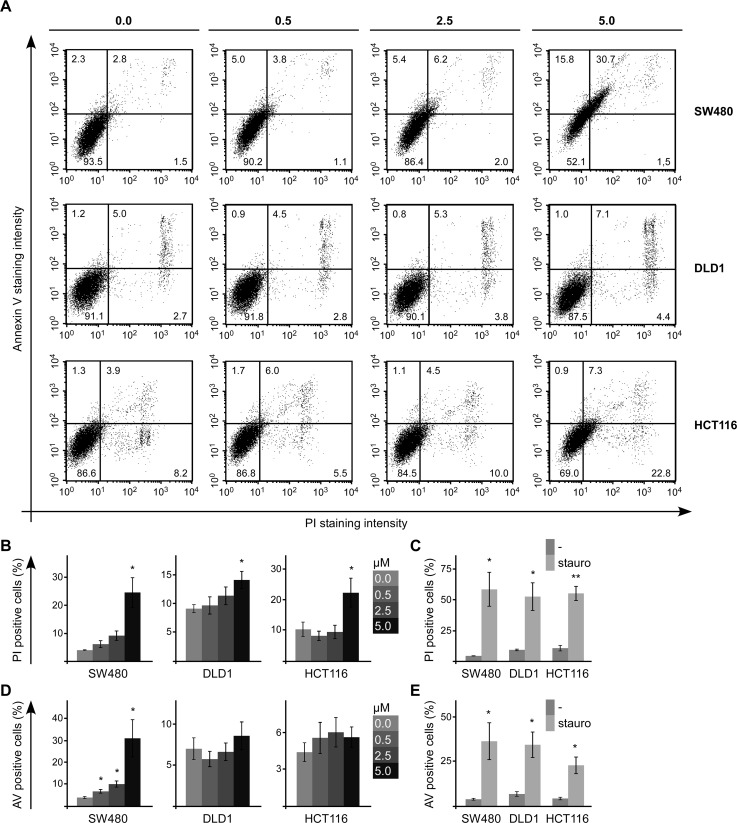
SFN induces death of colorectal cancer cells **(A)** FACS-based measurement of Annexin V (y-axis) and propidium iodide (PI, x-axis) staining intensity of SW480, DLD1 and HCT116 cells which were treated for 24 h with 0, 0.5, 2.5 and 5 μM SFN, as indicated. Numbers within individual quadrants present the percentages of cells. **(B, D)** Quantification of **(B)** propidium iodide (PI) positive cells (upper right and lower right quadrants) and **(D)** Annexin V (AV) positive cells (upper left and upper right quadrants) of four independent experiments as shown in **(A)**. **(C, E)** Quantification of **(C)** propidium iodide (PI) positive cells (upper right and lower right quadrants) and **(E)** Annexin V (AV) positive cells (upper left and upper right quadrants) without and with staurosporine treatment of SW480, DLD1 and HCT116 cells, of four independent experiments as shown in Supplementary Figure 2A. Results are mean +/− SEM (n=4). ^*^p<0.05, ^**^p<0.01 (Student's *t* test).

First, SFN treated colorectal cancer cells were stained with propidium iodide (PI) and Annexin V to label dead and apoptotic cells, respectively. FACS-based measurement of PI and Annexin V staining intensities showed that SFN treatment of SW480 cells for 24 h significantly increased the percentage of dead (PI-positive) and apoptotic (Annexin V-positive) cells in a dose-dependent manner indicating the induction of cell death by SFN via triggering apoptosis (Figure 2A, 2B, 2D). In DLD1 and HCT116 cells, SFN treatment induced cell death without significantly increasing the rate of apoptotic cells suggesting induction of cell death in an apoptosis-independent way (Figure 2A, 2B, 2D). In these experiments staurosporine was used as positive control showing that cell death as well as apoptosis can be efficiently induced in all three colorectal cancer cell lines (Figure 2C, 2E, Supplementary Figure 2A). FACS-based detection of SFN-induced apoptosis in SW480 cells could be confirmed via microscopic analysis of Annexin V and ethidium bromide (marks dead cells) stained cells (Supplementary Figure 2B). Moreover, quantification of the sub-G1 cells, which have less than one DNA equivalent due to DNA fragmentation in late apoptosis, showed that SFN treatment increased the fraction of these late-apoptotic cells in SW480 cells (Supplementary Figure 2C). This increase was less pronounced in DLD1 and HCT116 cells in line with the Annexin V staining results (Supplementary Figure 2C). The experiments suggest that SFN might induce cell death differentially in SW480 cells compared to DLD1 and HCT116 cells. However, SFN induced cell death in all three colorectal cancer cell lines.

**Figure 3:**
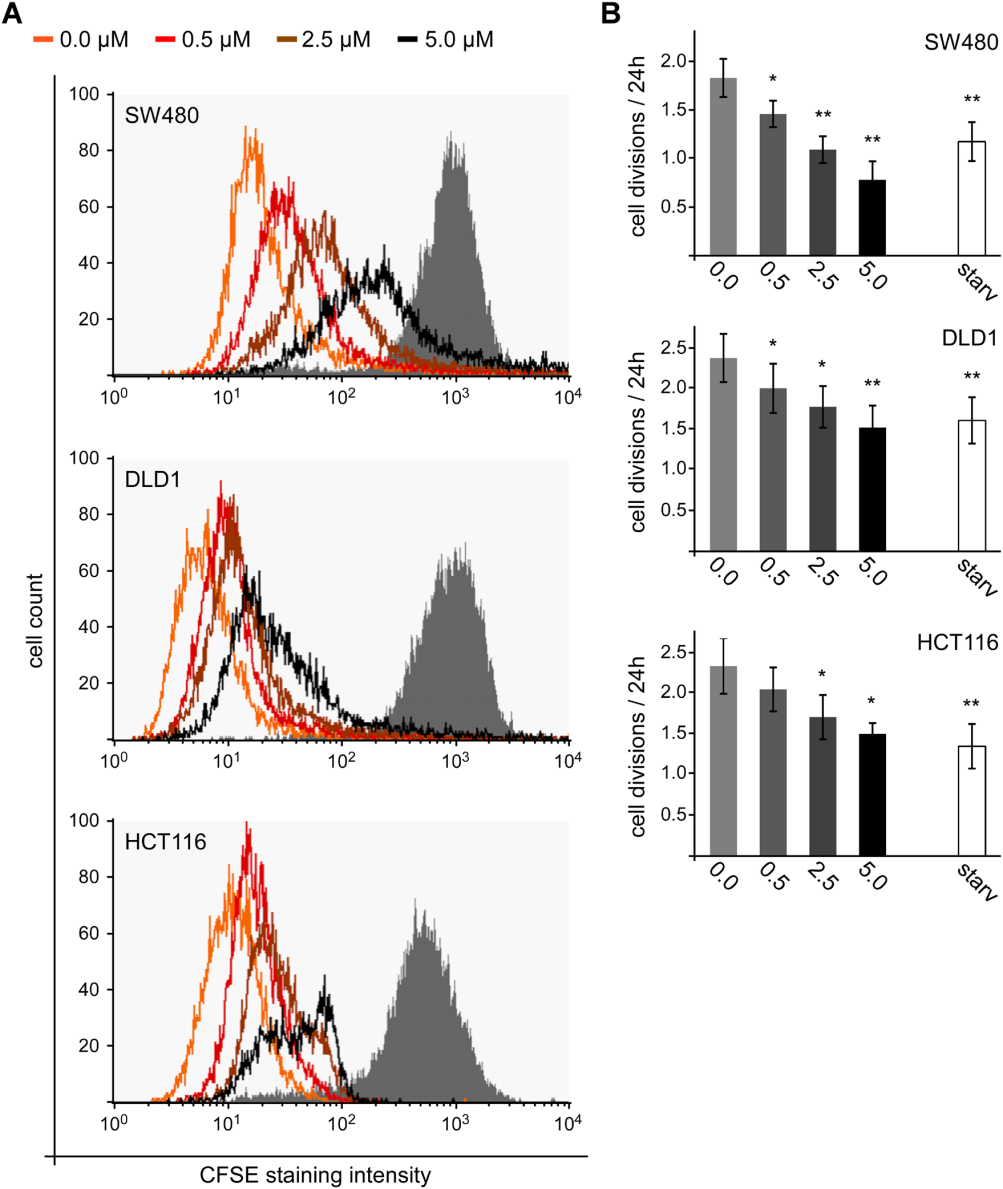
SFN inhibits proliferation of colorectal cancer cells **(A)** FACS-based measurement of CFSE staining intensity in SW480, DLD1 and HCT116 cells directly after the CFSE labeling pulse (grey filled) and after 72 h of treatment with indicated SFN concentrations (unfilled lines). **(B)** Calculated number of cell divisions per 24 h based on four independent CFSE dye dilution experiments as shown in **(A)**. Starvation (starv) from serum withdrawal was used as a positive control for reduced proliferation; respective curves are shown in Supplementary Figure 3. Results are mean +/− SEM (n=4). ^*^p<0.05, ^**^p<0.01 (Student's *t* test).

To directly assess cell proliferation, pulse-trace experiments with carboxyfluorescein succinimidyl ester (CFSE) were performed. After a labeling pulse with CFSE to couple this fluorescent dye to cellular macro molecules, the labelled cells were treated with different SFN concentrations (0, 0.5, 2.5 and 5 μM) for 72 h before FACS-based measurement of the remaining CFSE staining intensity. Every cell division reduces the CFSE staining intensity by 50% due to the equal distribution of the labelled macro molecules between both daughter cells. Importantly, the measured CFSE staining intensity after 72 h was significantly higher in SFN treated cells compared to untreated controls in SW480, DLD1 and HCT116 cells (Figure 3A). Moreover, there was a positive correlation between SFN concentrations and remaining CFSE staining indicating that SFN treatment inhibits cell proliferation in colorectal cancer cells in a dose-dependent manner. Since the CFSE staining is reduced by 50% with every cell division, the CFSE staining reduction within 72 h allowed to estimate the number of cell divisions. Of note, treatment with 5 μM SFN reduced the number of cell divisions in SW480, DLD1 and HCT116 cells by 57, 36 and 35%, respectively (Figure 3B). As a positive control for inhibition of proliferation, cells were grown in medium without serum for 72 h (Supplementary Figure 3). In the three cell lines, 5 μM SFN inhibited proliferation as efficiently (DLD1, HCT116) or even more efficiently (SW480) than withdrawal of serum growth factors (Figure 3B).

Together, our data suggest that SFN reduces numbers of colorectal cancer cells by induction of cell death as well as inhibition of cell proliferation.

### SFN inhibits Wnt/β-catenin signaling in colorectal cancer cells

**Figure 4:**
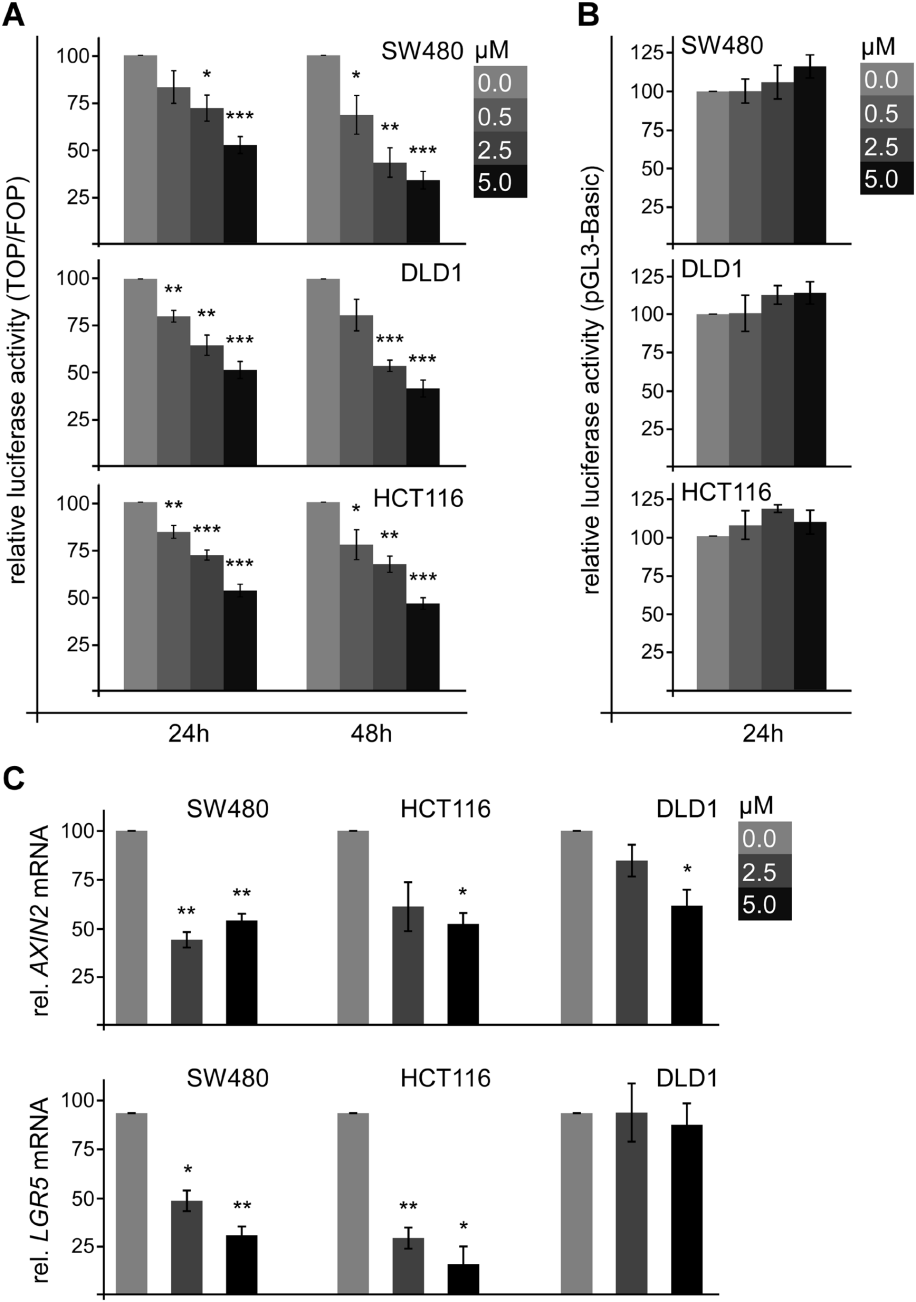
SFN inhibits Wnt/β-catenin signaling in colorectal cancer cells **(A)** Luciferase activity (TOP/FOP) in SW480, DLD1 and HCT116 cells which were treated with indicated SFN concentrations for 24 h and 48 h. Results are mean +/− SEM (n=5). ^*^p<0.05, ^**^p<0.01, ^***^p<0.001 (Student's *t* test). **(B)** Luciferase activity (pGL3-basic, β-catenin-independent) in SW480, DLD1 and HCT116 cells which were treated with indicated SFN concentrations for 24 h. Results are mean +/− SEM (n=3). **(C)** mRNA expression of the β-catenin target genes *AXIN2* (upper panel) and *LGR5* (lower panel) normalized to *GAPDH* in SW480, DLD1 and HCT116 cells which were treated with indicated SFN concentrations for 48 h. Results are mean +/− SEM (n=3). ^*^p<0.05, ^**^p<0.01 (Student's *t* test).

Wnt/β-catenin signaling activity is associated with cell survival and well-characterized to induce cell proliferation. Therefore, we determined whether SFN inhibits Wnt/β-catenin signaling to reduce proliferation of colorectal cancer cells. Wnt/β-catenin signaling activity was measured by the β-catenin-dependent TCF optimal (TOP)-FLASH luciferase reporter normalized to the Far from optimal (FOP) control reporter. Importantly, SFN treatment for 24 h significantly reduced the TOP-FLASH reporter activity in SW480, DLD1 and HCT116 cells in a dose-dependent manner (Figure 4A). Inhibition of Wnt/β-catenin signaling by SFN also correlated with the time of treatment as shown by stronger inhibition up to 70% after prolonged treatment (48 h, Figure 4A). SFN treatment did not affect the activity of a β-catenin-independent luciferase reporter (pGL3-Basic) demonstrating specific inhibition of β-catenin-dependent transcription by SFN (Figure 4B). Moreover, SFN treatment significantly reduced the mRNA expression of the bona fide β-catenin target genes *AXIN2* and *LGR5* in SW480 and HCT116 cells (Figure 4C) [7, 8]. In DLD1 cells, only *AXIN2* expression was significantly reduced (Figure 4C). *LGR5* expression might be co-stimulated independently of β-catenin in this cell line. These experiments characterize SFN as a potent inhibitor of Wnt/β-catenin signaling in colorectal cancer cells.

### SFN inhibits Wnt/β-catenin signaling downstream of β-catenin degradation

**Figure 5:**
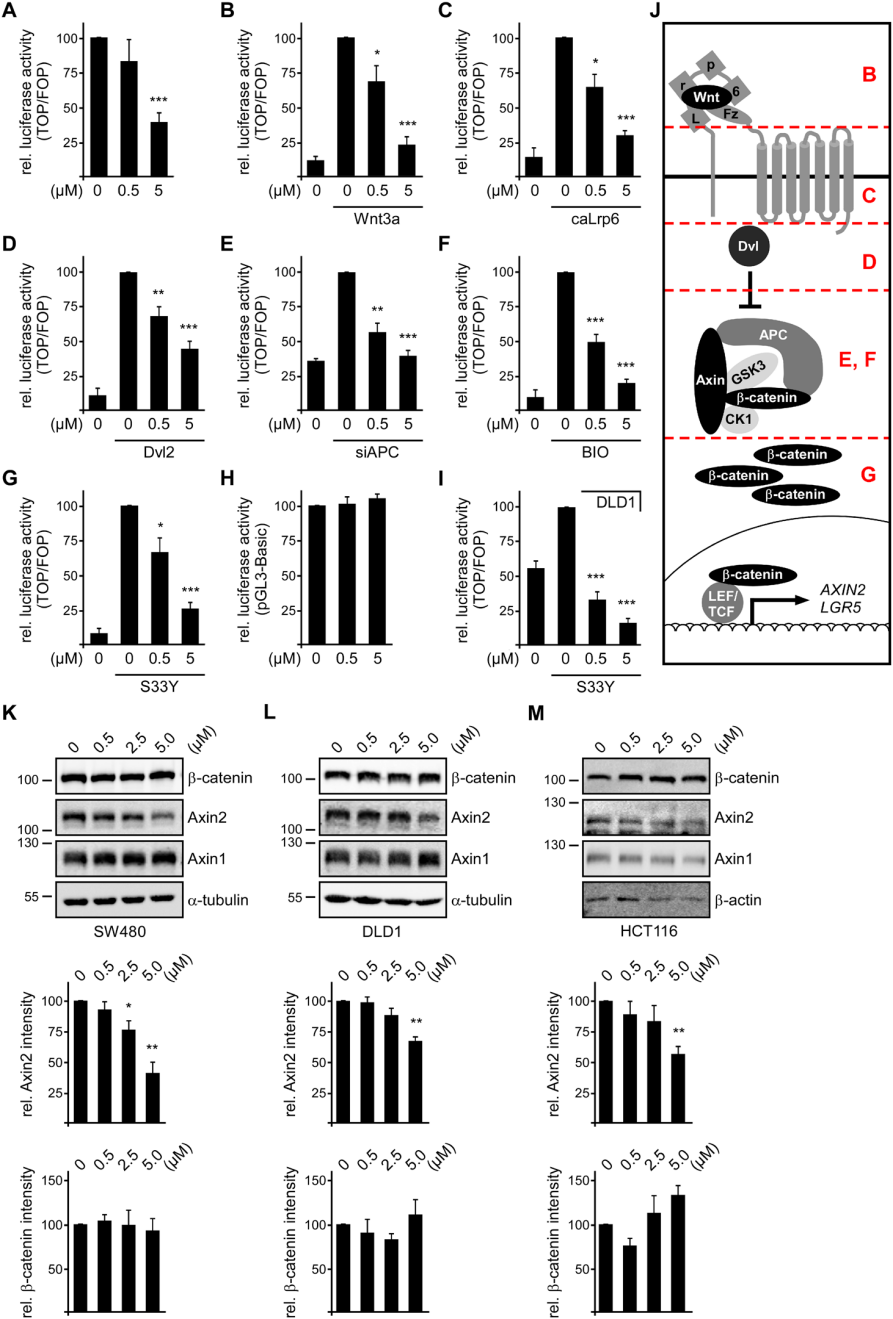
SFN inhibits Wnt/β-catenin signaling downstream of β-catenin degradation **(A-G)** Luciferase activity (TOP/FOP) after 24 h treatment with indicated SFN concentrations (0, 0.5, 5 μM) in HEK293T cells with basal Wnt signaling activity **(A)** or stimulated Wnt signaling activity by Wnt3a conditioned medium **(B)**, transient expression of constitutively active Lrp6 **(C)** or Dvl2 **(D)**, knockdown of APC **(E)**, GSK3 inhibition via BIO **(F)** or transient expression of the stabilized β-catenin mutant S33Y **(G)**. **(H)** Luciferase activity (pGL3-basic, β-catenin-independent) in HEK293T cells which were treated with indicated SFN concentrations for 24 h. **(I)** Luciferase activity (TOP/FOP) in DLD1 cells transiently expressing the stabilized β-catenin mutant S33Y, after treatment with indicated SFN concentrations for 24 h. Results are mean +/− SEM (n=5). ^*^p<0.05, ^**^p<0.01, ^***^p<0.001 (Student's *t* test). **(J)** Schematic illustration of the Wnt/β-catenin signaling cascade. Positions of pathway activation in **(B to G)** are indicated in red. **(K-M)** Upper panels: Western blotting for indicated proteins in hypotonic lysates of SW480 **(K)**, DLD1 **(L)** and HCT116 **(M)** cells which were treated for 24 h with SFN concentrations indicated above the blots. Middle panels: 2D densitometry quantification of Axin2 levels (β-catenin target gene) which were normalized to the expression of Axin1 (constitutively expressed Axin2 homolog) from four independent experiments as shown in the upper panels. Lower panels: 2D densitometry quantification of β-catenin levels which were normalized to the loading control (α-tubulin/β-actin) from four independent experiments as shown in the upper panels. Results are mean +/− SEM (n=4). ^*^p<0.05, ^**^p<0.01 (Student's *t* test).

In breast cancer cells, SFN was described to inhibit Wnt/β-catenin signaling by activating GSK3 [12]. Since β-catenin phosphorylation in colorectal cancer cells is impaired due to APC truncations or impossible due to β-catenin mutations, it is unlikely that increased GSK3 activity can be accounted for inhibition of Wnt/β-catenin signaling by SFN in SW480, DLD1 and HCT116 cells. However, to formally exclude this mechanism in colorectal cancer cells we determined whether SFN treatment reduces the inhibiting phosphorylation of GSK3β at serine 9, as described for breast cancer cells [12]. Western blot analysis revealed that phosphorylation of GSK3β at serine 9 was not reduced but rather increased upon SFN treatment in colorectal cancer cells (Supplementary Figure 4A-4C). To reveal other mechanisms for Wnt/β-catenin signaling inhibition by SFN we started to pinpoint at which level of the Wnt/β-catenin signaling cascade SFN interferes (Figure 5J). For this, we used various stimuli to activate Wnt/β-catenin signaling in HEK293T cells transfected with the TOP-FLASH reporter and analyzed whether signaling activity could be blocked by SFN. Although the Wnt/β-catenin activity in unstimulated HEK293T cells is rather low, it was significantly reduced by SFN demonstrating functionality of SFN in HEK293T cells (Figure 5A). Activation of Wnt signaling at the receptor level by Wnt3a conditioned medium or transfection of a constitutively active Lrp6 co-receptor mutant (caLrp6) was efficiently counteracted by SFN treatment (Figure 5B, 5C, 5J). Likewise, activation of the pathway by transient Dvl2 expression, a positive regulator involved in signal transduction from the receptors to the β-catenin destruction complex, was blocked by SFN (Figure 5D, 5J). Also, Wnt/β-catenin signaling activated by direct inhibition of the β-catenin destruction complex via siRNA mediated knockdown of APC, which mimics the APC loss of function in SW480 and DLD1 cells, or via the GSK3 inhibitor BIO was prevented by SFN treatment (Figure 5E, 5F, 5J). Finally, even activation of the pathway downstream of the β-catenin destruction complex by transient expression of a non-degradable β-catenin mutant (S33Y) was decreased by SFN (Figure 5G, 5J). We verified by Western blotting that SFN treatment did not reduce the levels of transiently expressed caLrp6, Dvl2 and β-catenin S33Y (Supplementary Figure 5A-5C). As seen in colorectal cancer cells, SFN did not affect expression of a β-catenin-independent reporter (pGL3-Basic) in HEK293T cells showing specific inhibition of β-catenin signaling by SFN (Figure 5H).

Inhibition of Wnt/β-catenin signaling in β-catenin S33Y expressing HEK293T cells is in line with inhibition of signaling in HCT116 cells which harbor a similar stabilizing β-catenin mutation. Moreover, SFN was also able to inhibit β-catenin S33Y-activated Wnt signaling in DLD1 cells indicating that SFN functions by a common mechanism in HEK293T cells and colorectal cancer cells (Figure 5I, Supplementary Figure 5D). Finally, SFN treatment of colorectal cancer cells did not decrease β-catenin levels in hypotonic cell extracts (Figure 5K-5M). Efficient inhibition of Wnt/β-catenin signaling in these experiments is shown by the decrease of the β-catenin target gene Axin2 in contrast to its constitutively expressed homolog Axin1 (Figure 5K-5M).

### SFN induces the formation of inactive β-catenin-containing transcription complexes

**Figure 6:**
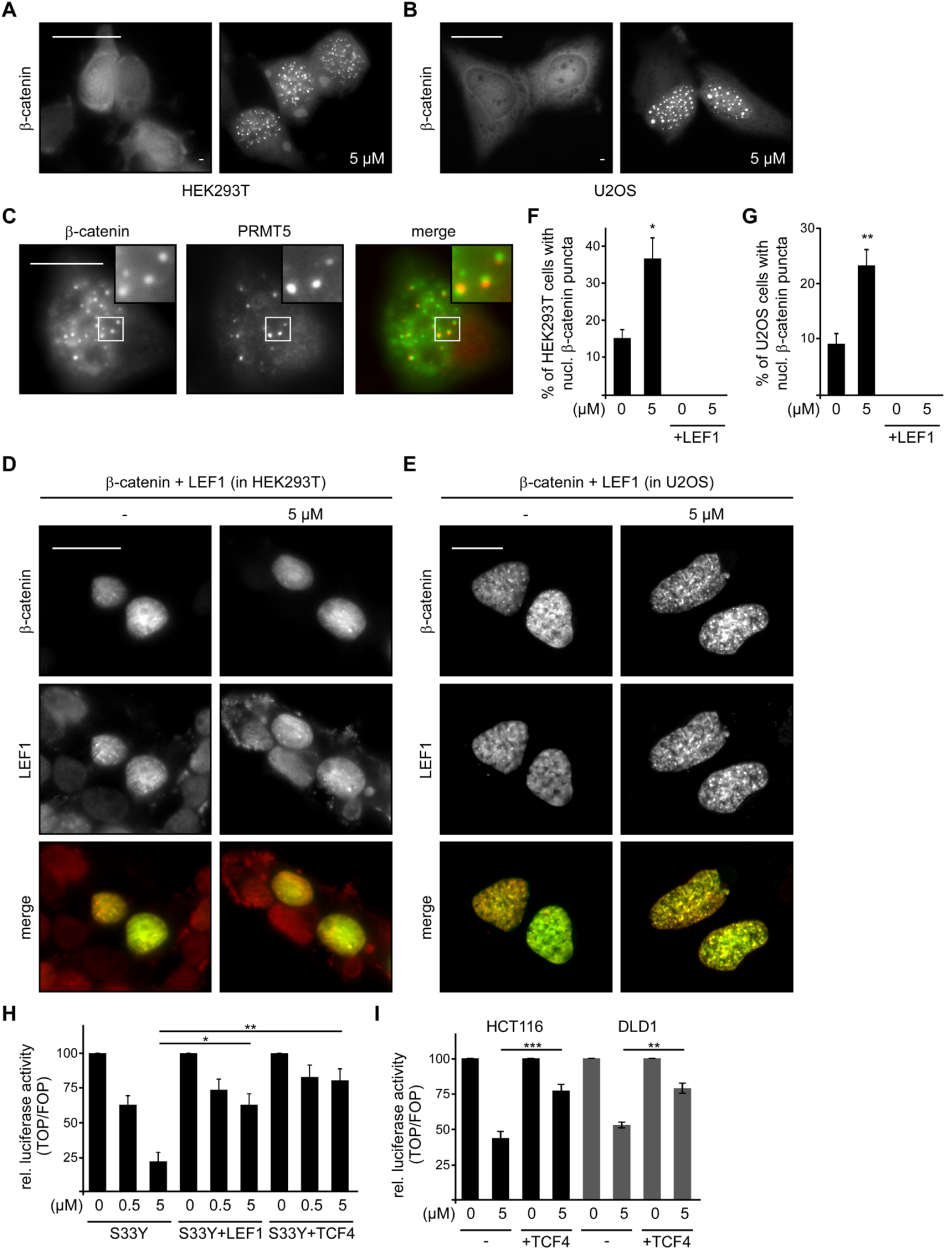
SFN induces the formation of inactive β-catenin-containing transcription complexes **(A** and **B)** GFP-β-catenin fluorescence in transfected HEK293T **(A)** and U2OS cells **(B)** which were left untreated (−) or treated with 5 μM SFN for 24 h. Scale bar: 20 μm. **(C)** Immunofluorescence staining for PRMT5 (red) in GFP-β-catenin (green) transfected HEK293T cells which were treated with 5 μM SFN for 24 h. Scale bar: 10 μm. Insets are magnified in the top right corner. **(D** and **E)** GFP-β-catenin fluorescence (green) and immunofluorescence staining for HA-LEF1 (red) in co-transfected HEK293T **(D)** and U2OS cells **(E)**. Cells were left untreated (−) or treated with 5 μM SFN for 24 h. Scale bar: 20 μm. **(F** and **G)** Quantification of cells with nuclear (nucl.) β-catenin puncta from experiments as described in **(A** and **D) (F)**, and **(B** and **E) (G)**. Results are mean +/− SEM (n=3). ^*^p<0.05, ^**^p<0.01 (Student's *t* test). **(H)** Luciferase activity (TOP/FOP) after 24 h treatment with indicated SFN concentrations (0, 0.5, 5 μM) in HEK293T cells transfected with the stabilized β-catenin mutant S33Y, S33Y + HA-LEF1 or S33Y + CFP-TCF4. Results are mean +/− SEM (n=5). ^*^p<0.05, ^**^p<0.01 (Student's *t* test). **(I)** Luciferase activity (TOP/FOP) after 24 h treatment with indicated SFN concentrations (0, 5 μM) in HCT116 (black bars) and DLD1 cells (grey bars) without (−) or with co-transfection of CFP-TCF4. Results are mean +/− SEM (n=5). ^**^p<0.01, ^***^p<0.001 (Student's *t* test).

Together, these experiments suggest that SFN inhibits Wnt/β-catenin signaling downstream of β-catenin degradation at the level of nuclear import or activation of target genes in the nucleus. To investigate this further, the nuclear localization of β-catenin after SFN treatment was assessed. Transiently expressed β-catenin is localized diffusely throughout the cytoplasm and the nucleus, and sporadically forms nuclear puncta in a low percentage of cells. Importantly, SFN treatment significantly increased the percentage of cells showing these β-catenin puncta as well as the size of these puncta in different cell lines (Figure 6A, 6B, 6F, 6G). These puncta were reminiscent of previously described nuclear β-catenin puncta which have been linked to inhibition of Wnt/β-catenin signaling and are enriched for the transcriptional repressor Protein arginine N-methyltransferase 5 (PRMT5) [20, 21]. Therefore, we performed co-staining for PRMT5. Indeed, in some cells with nuclear β-catenin puncta we observed partial, but clear co-localization of β-catenin and endogenous PRMT5 in these puncta (Figure 6C). Co-localization with the transcriptional repressor PRMT5 suggests that these nuclear β-catenin accumulations are transcriptionally inactive and composition-wise similar to those described previously [20].

Upon co-expression of the transcription factor LEF1, which is known to associate with β-catenin to activate β-catenin-dependent transcription, β-catenin was completely recruited into the nucleus, as previously reported (Figure 6D, 6E) [6]. Of note, SFN failed to induce the formation of β-catenin puncta in the presence of LEF1 (Figure 6D-6G). In line, co-expression of LEF1 or TCF4, another member of the TCF/LEF transcription factor family, together with β-catenin S33Y largely rescued the inhibition of β-catenin-dependent transcription by SFN in luciferase reporter assays (Figure 6H). Also in colorectal cancer cells, transient expression of TCF4 significantly rescued SFN-induced inhibition of Wnt/β-catenin signaling (Figure 6I). Our results suggest that SFN inhibits Wnt/β-catenin signaling by inducing the formation of nuclear β-catenin puncta which might represent inactive or even repressive β-catenin containing transcription complexes (see Discussion).

## DISCUSSION

In this study, we characterize SFN as potent inhibitor of Wnt/β-catenin signaling in colorectal cancer cells. SFN inhibited β-catenin-dependent reporter activity and repressed β-catenin target gene expression in a dose-dependent manner at concentrations which can be achieved in the blood through oral SFN uptake [15]. To our knowledge, this is the first time to show inhibition of Wnt/β-catenin signaling by SFN in cells with mutations in components of the β-catenin destruction complex (APC; SW480, DLD1) or stabilizing mutations of β-catenin (HCT116). So far, only few studies describe inhibition of Wnt/β-catenin signaling by SFN in cells with an intact β-catenin degradation machinery and the underlying mechanism(s) are poorly understood. Inhibition of Wnt signaling by enhancing GSK3 function, as has been described for SFN in breast cancer cells, is unlikely to be effective in colorectal cancer cells and could not be revealed in our study [12]. In contrast, our data clearly show SFN-induced inhibition of Wnt/β-catenin signaling downstream of β-catenin degradation because (i) SFN treatment did not reduce β-catenin levels, (ii) SFN inhibited Wnt signaling activated by the stabilized β-catenin mutant S33Y, (iii) SFN inhibited Wnt signaling in HCT116 cells which harbor a stabilizing β-catenin mutation similar to the S33Y mutant, and (iv) inhibition of Wnt signaling by SFN could be rescued by LEF1 or TCF4 expression. Inhibition of Wnt/β-catenin signaling downstream of β-catenin degradation explains how SFN can be functional in colorectal cancer cells with impaired β-catenin degradation.

Mechanistically, SFN, most likely, prevents the formation of active β-catenin-based transcription complexes in the nucleus. Like ARID1B, a chromatin remodeling factor which inhibits Wnt/β-catenin signaling, SFN increased the formation of nuclear β-catenin puncta, which had previously been linked to closed chromatin structures and reduced β-catenin-dependent transcription [20]. Increasing the levels of activating transcription factors of the TCF/LEF family (LEF1, TCF4) by transient expression rescued the induction of inhibitory β-catenin puncta as well as the inhibition of Wnt/β-catenin signaling by SFN. Possibly, SFN treatment causes clustering of β-catenin in presumably inactive puncta, and/or induces binding of negative chromatin remodeling factors such as PRMT5 to β-catenin or other complex components. A nuclear function of SFN, as suggested by our proposed mechanism, is in line with a previous study demonstrating that SFN inhibits NF-κB signaling in the nucleus downstream of IκB [22].

The Wnt/β-catenin signaling pathway is known to promote cell survival and to stimulate cell proliferation. In line, after inhibition of Wnt/β-catenin signaling by SFN we observed increased cell death and reduced proliferation in all three colorectal cancer cell lines. Although inhibition of Wnt/β-catenin signaling could explain the induction of cell death by SFN [23, 24], there might be contributions via other mechanism(s), like e.g. SFN-induced apoptosis via increased Erk1/2 phosphorylation, which was reported for non-small cell lung cancer cells [25]. Together, increased cell death and reduced proliferation resulted in a dramatic reduction of cell numbers upon SFN treatment in colony formation assays and MTT-based cell growth assays *in vitro*. The observed growth inhibition of colorectal cancer cells by SFN is in line with previous studies [19]. An early *in vivo* study showed inhibition of intestinal tumorigenesis by SFN in the APC^MIN^ mouse model [18]. The authors excluded inhibition of Wnt/β-catenin signaling because β-catenin levels were not altered [18]. Our data show that SFN in fact inhibits Wnt signaling without altering β-catenin levels suggesting that inhibition of Wnt signaling could well contribute to the inhibition of intestinal tumorigenesis *in vivo*.

By revealing SFN-mediated inhibition of Wnt/β-catenin signaling in colorectal cancer cells, our study significantly adds to the understanding of how SFN can inhibit colorectal cancer growth. Considering cancer treatment or prevention, it is important to know the mechanistic function of drugs or natural compounds because it helps to predict whether individual tumors will be responsive or not. Since hyperactive Wnt/β-catenin signaling represents a hallmark of human colorectal cancerogenesis with more than 90% of the cancers exhibiting genetic alteration activating the pathway [2], inhibition of Wnt signaling by SFN suggests broad responsiveness of colorectal cancers to SFN treatment and benefits for colorectal cancer prevention through SFN uptake.

## MATERIALS AND METHODS

### Cell culture, transfection and small chemicals

CX-1, DLD1, HCT116, HEK293T, SW48, SW480, U2OS and WiDr cells were grown in DMEM supplemented with 10% fetal bovine serum (FBS) and penicillin/streptomycin in a 10% CO_2_ atmosphere at 37°C, and subcultured according to ATCC recommendations. DLD1 and SW480 cells were transfected with Lipofectamine2000 (Invitrogen) and HCT116, HEK293T and U2OS cells with polyethylenimine, according to manufacturers' instructions. Wnt3a-conditioned medium was prepared as described previously [26]. SFN and BIO were obtained from Sigma-Aldrich.

### MTT cell growth assay

Two thousand cells (DLD1 and HCT116) or 3000 cells (SW480) were seeded per well in 96-well flat-bottomed tissue culture plates and grown in the presence of different SFN concentrations for 24 h to 72 h, as indicated in Figure 1A. At the end of every time point, MTT (Sigma-Aldrich) was added at a final concentration of 0.5 mg/ml and cells were incubated for another 4 h at 37°C to allow MTT cleavage within the living cells. Afterwards, the produced MTT formazan was dissolved by adding 100 μl isopropanol with 0.04 N HCl per well and the homogenous violet solution was measured with a Spectra MAX 190 (Molecular Devices) at 570 nm and normalized to the measurement at a reference wavelength of 690 nm. The measured formazan absorbance is directly proportional to the number of living cells. The assay was performed in technical quadruplicates.

### Colony formation assay

For colony formation assays, 2500 CX-1, DLD1, HCT116, SW48, SW480, U2OS or WiDr cells were seeded per well of a 6-well plate and grown in the presence of different SFN concentrations for 96 h. Afterwards, cells were fixed with 3% PFA for 10 min at RT, stained with ethidium bromide (50 μg/ml in PBS) and visualized in a UV gel documentation system (Herolab). Images were acquired at constant settings. Using the Metamorph analysis software (Carl Zeiss), numbers and sizes of colonies were quantified from these images in an automated fashion by applying a threshold for light objects.

### FACS-based measurement of cell death and proliferation

To assess SFN induced cell death, cells were treated with indicated SFN concentrations for 24 h or with 1 μM staurosporine (Sigma-Aldrich) overnight, collected in FACS buffer (1x PBS with 2% FBS and 5 mM EDTA) and stained with 5 μg/ml propidium iodide (Sigma-Aldrich) and Annexin V-FITC (BD Bioscience). Red fluorescence intensity (propidium iodide) or green fluorescence intensity (Annexin V-FITC) of 10,000 individual cells was measured at a FACSCalibur (BD Bioscience). Cell debris was excluded from the analysis by gating on forward and side scatter.

For quantification of the apoptotic sub-G1 cells, cells were either left untreated or treated with 5 μM SFN for 24 h, collected, fixed in cold 80% ethanol at 4°C and stained with propidium iodide (5 μg/ml propidium iodide and 0.4 mg/ml RNase A in PBS). Red fluorescence intensity (propidium iodide) of 10,000 individual cells was measured at a FACSCalibur (BD Bioscience).

To assess cell proliferation, cells were pulse labeled by incubation with 5 μM CFSE (Sigma-Aldrich) for 20 min and either directly measured after the pulse or grown for 72 h in the presence of different SFN concentrations or in serum free medium (starvation). Green fluorescence intensity (CFSE) of 10,000 individual cells was measured at a FACSCalibur (BD Bioscience). Cell debris was excluded from the analysis by gating on forward and side scatter.

### Microscopic analysis of cell death

SW480 cells were treated with 5 μM SFN for 48 h after which attached cells were trypsinized and combined with the floating cells, pelleted by centrifugation (300 g, 3 min, RT) and resuspended in staining solution (1x PBS with 5 μg/ml ethidium bromide [Carl Roth] and Annexin V-FITC [BD Bioscience]). After 15 min incubation at RT in the dark, 15 μl of the stained cell suspension was pipetted on a microscope slide, covered with a coverslip and immediately analyzed with an Axioplan II microscope system (Carl Zeiss). Images were acquired using MetaMorph analysis software (Carl Zeiss).

### Luciferase reporter assay

As previously described [27], cells were transfected with a luciferase expression plasmid either with a β-catenin-dependent promoter (TOP) or with a constitutive promoter (FOP), together with a β-galactosidase expression plasmid. Cells were co-transfected with further expression plasmids of interest or treated with different substances for 24 to 48 h, as stated in the figure legends. Afterwards, the cells were lysed in luciferase assay buffer (25 mM Tris-HCl pH8, 2 mM EDTA, 5% glycerol, 1% Triton X-100, 20 mM DTT) and the luciferase activity was measured by the emission of light upon oxidative luciferin decarboxylation with a Centro LB 960 Microplate Luminometer (Berthold technologies). The β-galactosidase activity was determined as release of yellow ortho-Nitrophenol upon ortho-Nitrophenyl-β-galactoside hydrolysis with a Spectra MAX 190 (Molecular Devices). Luciferase activities of TOP and FOP samples were normalized to the respective β-galactosidase activity to eliminate transfection efficiency-based differences before calculating TOP/FOP ratios. TOP/FOP assays were performed in technical duplicates.

### qRT-PCR analysis

Cells were treated with indicated SFN concentrations for 48 h before the whole cellular RNA was isolated using the RNeasy mini kit (QIAGEN) according to manufacturer's instructions. cDNA was synthesized with the AffinityScript QPCR cDNA Synthesis Kit (Agilent Technologies). Quantitative PCR for *AXIN2* (for: CCTCAGAGCGATGGATTTCGGG; rev: CCAGTTCCTCTCAGCAATCGGC), *LGR5* (for: CTTCCAACCTCAGCGTCTTCACC; rev: GTCAGA GCGTTTCCCGCAAGAC) and *GAPDH* (for: GTCAA GGCTGAGAACGGGAAGC; rev: GGACTCCACG ACGTACTCAGCG) was performed in a CFX96 Real-Time System (Bio-Rad) in triplicates. mRNA expression of the Wnt/β-catenin target genes *AXIN2* and *LGR5* was normalized to the housekeeping gene *GAPDH*.

### Cell lysis and Western blot

For Western blotting, cells were lysed after 24 h treatment with indicated SFN concentrations in hypotonic extraction buffer (20 mM Tris-HCl pH7.5, 1 mM EDTA, Roche protease inhibitor cocktail) (Figure 5, Supplementary Figure 4) or luciferase assay buffer (Supplementary Figure 5). Proteins were separated according to sizes by SDS-PAGE and transferred to a nitrocellulose membrane, which was probed with primary antibodies: m α β-catenin (sc-7963) (SantaCruz), rb α Axin1 (C76H11), rb α Axin2 (76G6), rb α GSK3β (27C10), rb α phospho-GSK3β (D85E12) (CellSignaling), rat α α-tubulin (MCA77G) (Serotec), m α β-actin (A5441), rb α HA (H6908) (Sigma-Aldrich), m α GFP (11814460001) (Roche), rb α RFP (ab62341) (Abcam). The horseradish peroxidase (HRP) activity of secondary antibodies, goat α mouse-HRP and goat α rabbit-HRP (Jackson ImmunoResearch), was detected with a LAS-3000 (FUJIFILM). Intensities of Western blot bands were quantified with AIDA 2D densitometry.

### Immunofluorescence

Cells were treated with SFN concentrations indicated in Figure 6 for 24 h before immunofluorescence staining was performed as described previously [28]. In short, cells were fixed in 100% methanol at −20°C or in 3% PFA at RT (for PRMT5 staining only), permeabilized with 0.5% Triton X-100 and blocked with medium to prevent unspecific antibody binding. Afterwards, cells were incubated with primary antibodies against indicated proteins (rb α PRMT5, ab31751, Abcam / rb α HA, H6908, Sigma-Aldrich) and fluorochrome-conjugated secondary antibody (goat α rabbit-Cy3, Jackson ImmunoResearch) for 1 h each. Stained cells were analyzed with an Axioplan II microscope system (Carl Zeiss) and images were acquired using MetaMorph analysis software (Carl Zeiss).

### Plasmids and siRNA

Expression plasmids for HA-Dvl2, RFP-caLrp6, YFP-β-catenin S33Y, GFP-β-catenin, HA-LEF1 and CFP-TCF4, and the siRNA against APC (5´-AAGACGUUGCGAGAAGUUGGA-3`) have been described previously [6, 28–31].

## SUPPLEMENTARY MATERIALS FIGURES


