# Supplementary material for: Sulforaphane inhibits growth and blocks Wnt/β-catenin signaling of colorectal cancer cells
Source: Oncotarget. 2018 Sep 21;9(74):33982–94. doi: 10.18632/oncotarget.26125 (PMC6188060; doi:10.18632/oncotarget.26125)
Supplement: Supplementary file 1 [file oncotarget-09-33982-s001.pdf]

Sulforaphane inhibits growth and blocks Wnt/β-catenin signaling of colorectal cancer cells

SUPPLEMENTARY MATERIALS

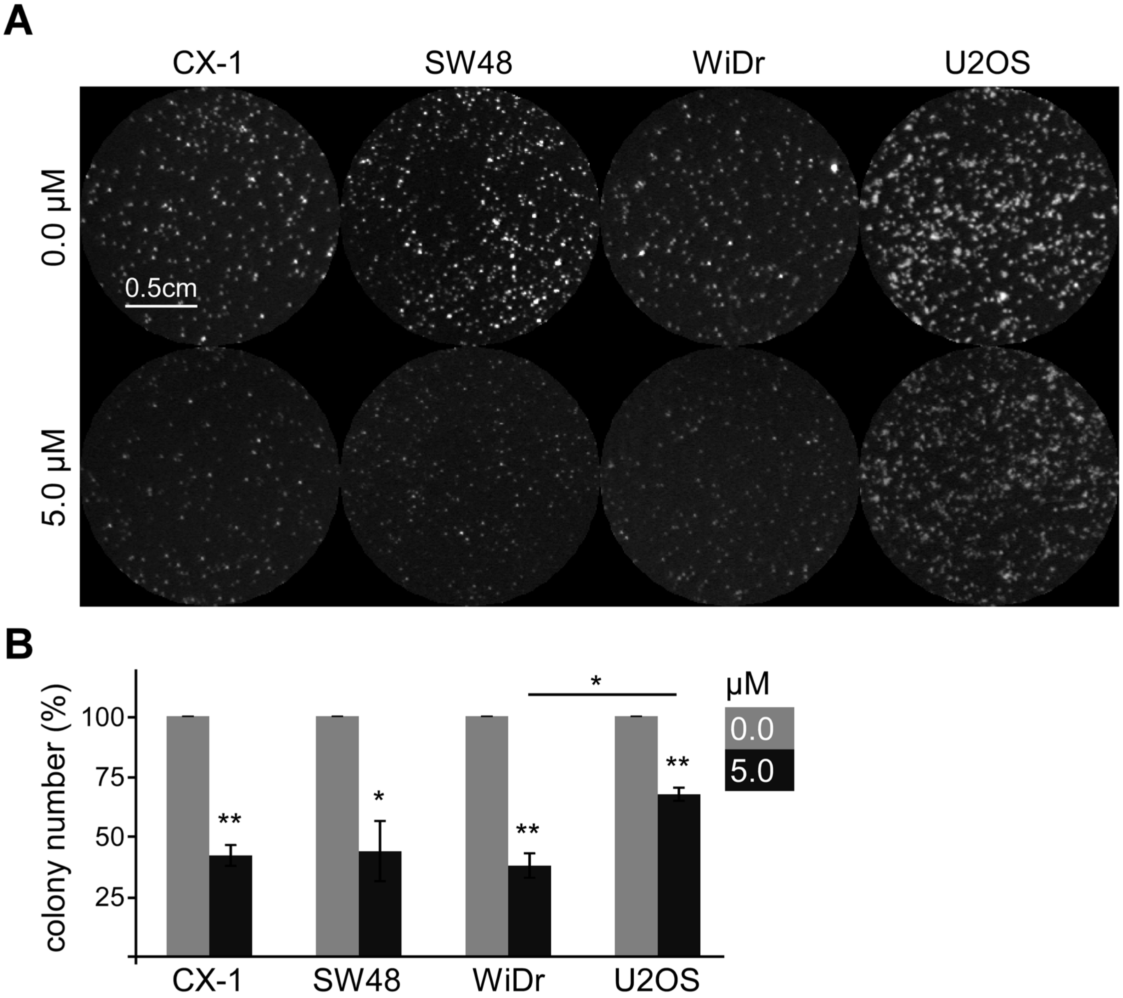

Supplementary Figure 1: SFN inhibits colony formation of colorectal cancer cells. (A) Cell colonies grown for 96 h from individual CX-1, SW48, WiDr, or U2OS cells in the presence of indicated SFN concentrations. Cells were stained by ethidium bromide incorporation and visualized with UV light. **(B)** Automated quantification of colony numbers from three independent experiments as in **(A)**. Results are mean +/- SEM (n=3). $^{*}p<0.05$, $^{**}p<0.01$ (Student's *t* test).

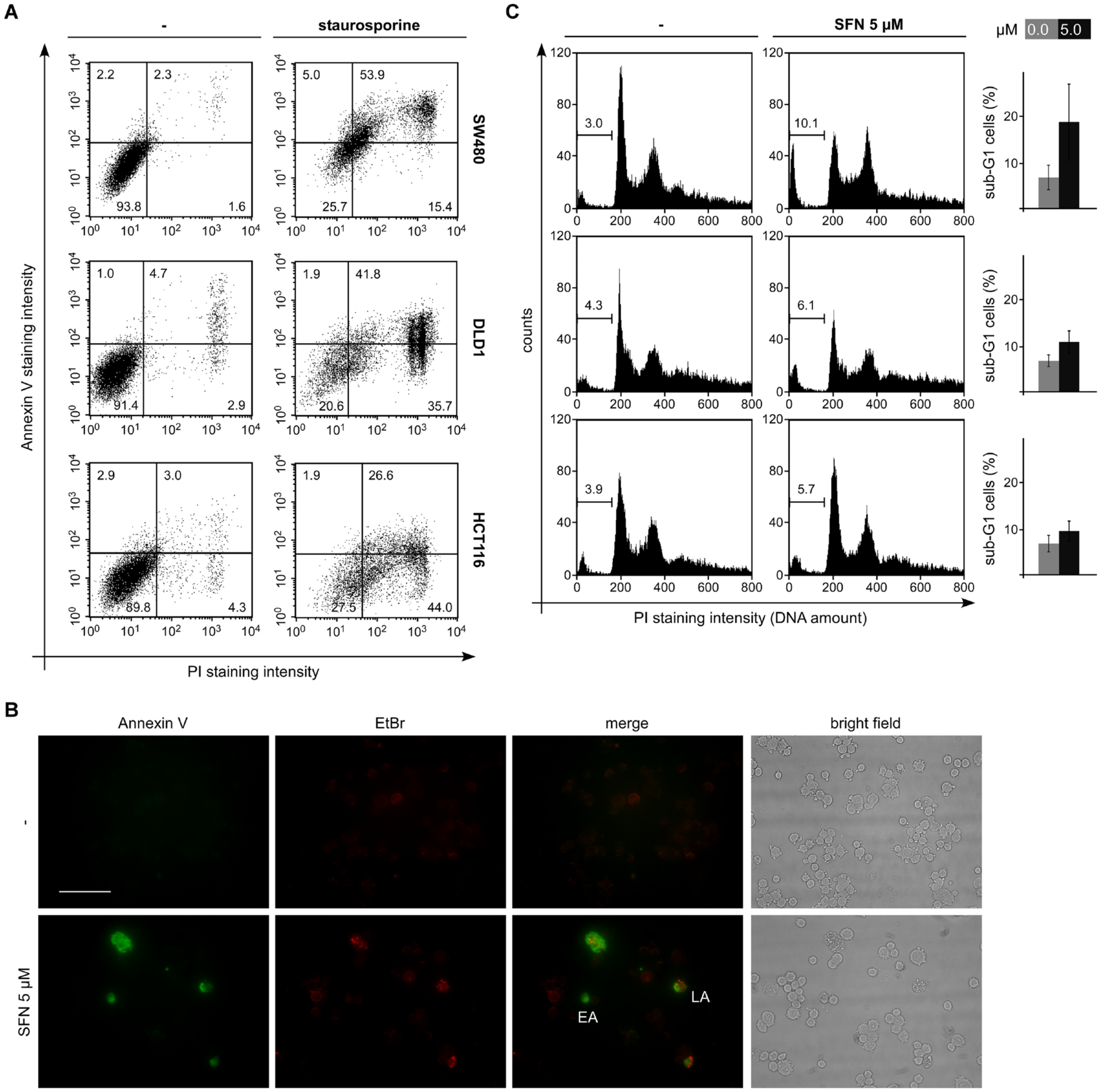

Supplementary Figure 2: Induction of cell death by staurosporine and SFN in colorectal cancer cells. (A) FACS-based measurement of Annexin V (y-axis) and propidium iodide (PI, x-axis) staining intensity of SW480, DLD1 and HCT116 cells without and with staurosporine treatment overnight, as indicated above. Numbers within individual quadrants present the percentages of cells. Quantification of four independent experiments is shown in Figure 2C, 2E. **(B)** Annexin V-FITC (green) and ethidium bromide (EtBr, red) fluorescence of stained SW480 cells which were left untreated (-) or treated with 5 µM SFN for 48 h. An early apoptotic cell (EA, only Annexin V positive) and a late apoptotic cell (LA, Annexin V and EtBr positive) are indicated. Scale bar: 100 µm. **(C)** Histograms show FACS-based numbers of cells (counts, y-axis) with different propidium iodide (PI, x-axis) staining intensities reflecting the DNA amount, of SW480 (upper row), DLD1 (middle row) and HCT116 cells (lower row) without and with 5 µM SFN treatment for 24 h. The fraction of cells with less than one DNA equivalent (sub-G1 cells) is indicated by the markers, and the numbers above these markers represent the percentage of cells in this fraction. Bar diagrams show the percentage of sub-G1 cells as mean +/- SEM of four independent experiments, as shown in the histograms (n=4).

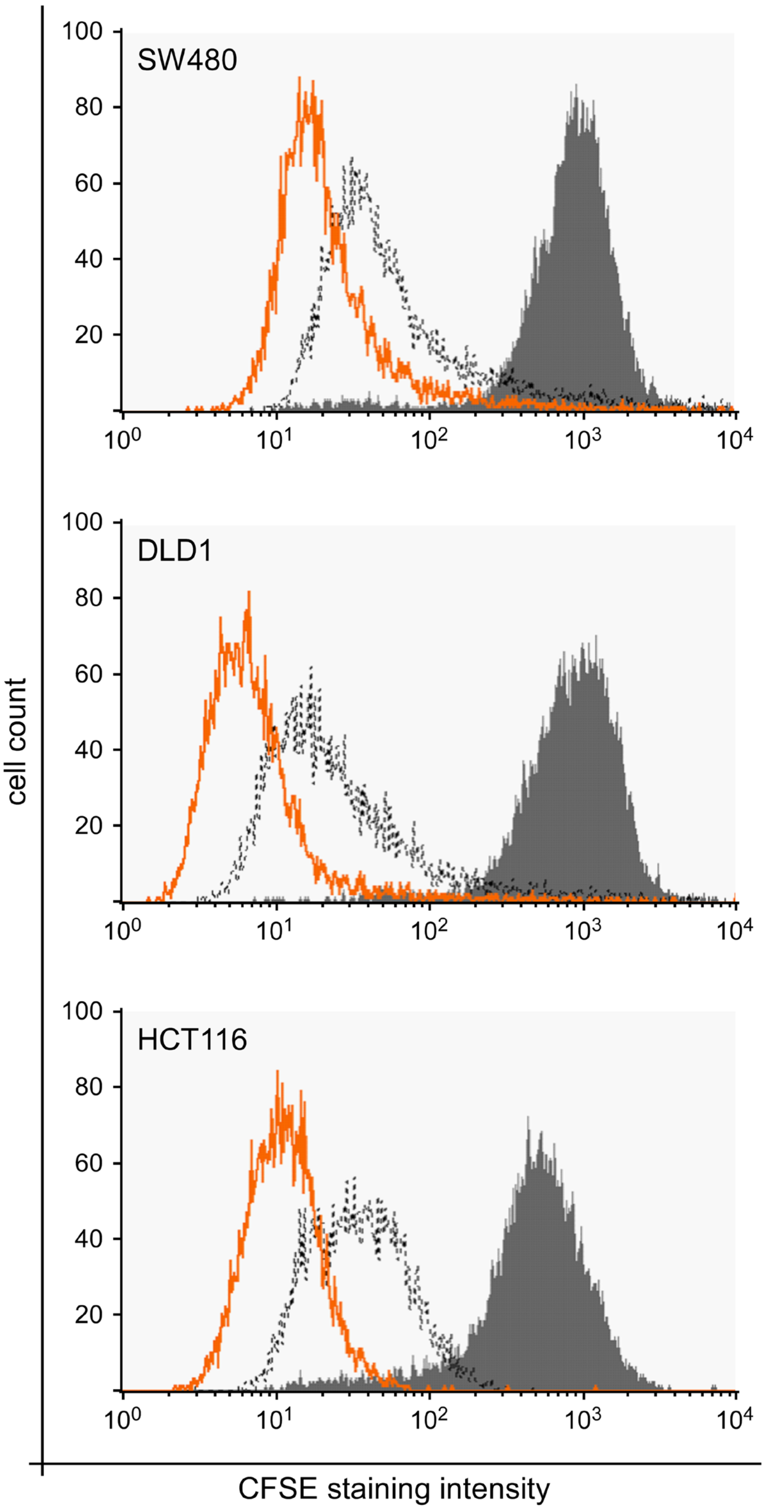

Supplementary Figure 3: Starvation inhibits proliferation of colorectal cancer cells. FACS-based measurement of CFSE staining intensity in SW480, DLD1 and HCT116 cells directly after the CFSE labeling pulse (grey, filled) and after 72 h without treatment (orange line) or with starvation (black dotted line). "Labelling pulse" and "without treatment" are from the same experiments as shown in Figure 3.

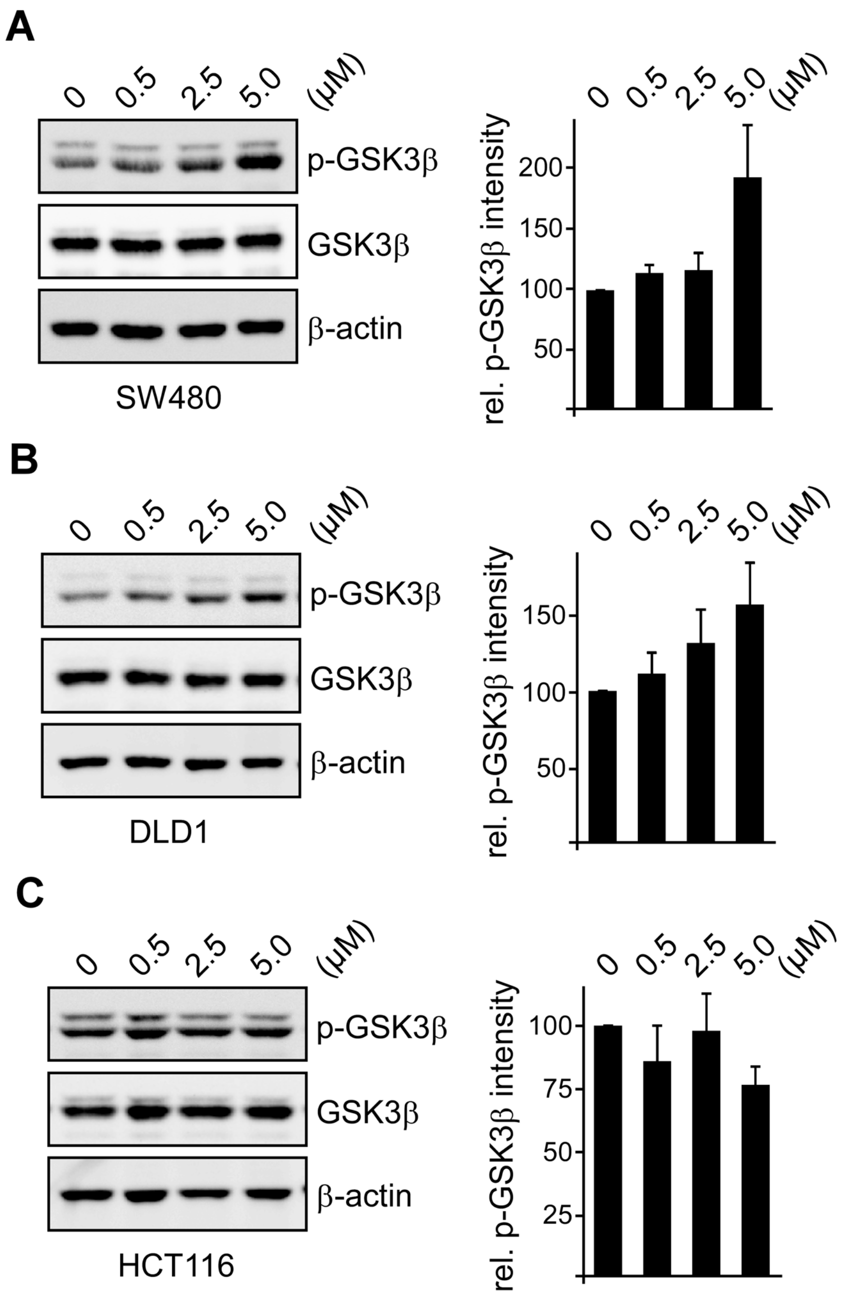

Supplementary Figure 4: SFN does not decrease GSK3β phosphorylation in colorectal cancer cells. (A-C) Left panel: Western blotting for phospho (p)-GSK3β, GSK3β and β-actin in lysates of SW480 **(A)**, DLD1 **(B)** and HCT116 **(C)** cells which were treated with SFN concentrations indicated above the blots for 24 h. Right panel: p-GSK3β band intensities were determined by 2D densitometry of three independent experiments as shown on the left and normalized to GSK3β intensities. Results are mean +/- SEM (n=3).

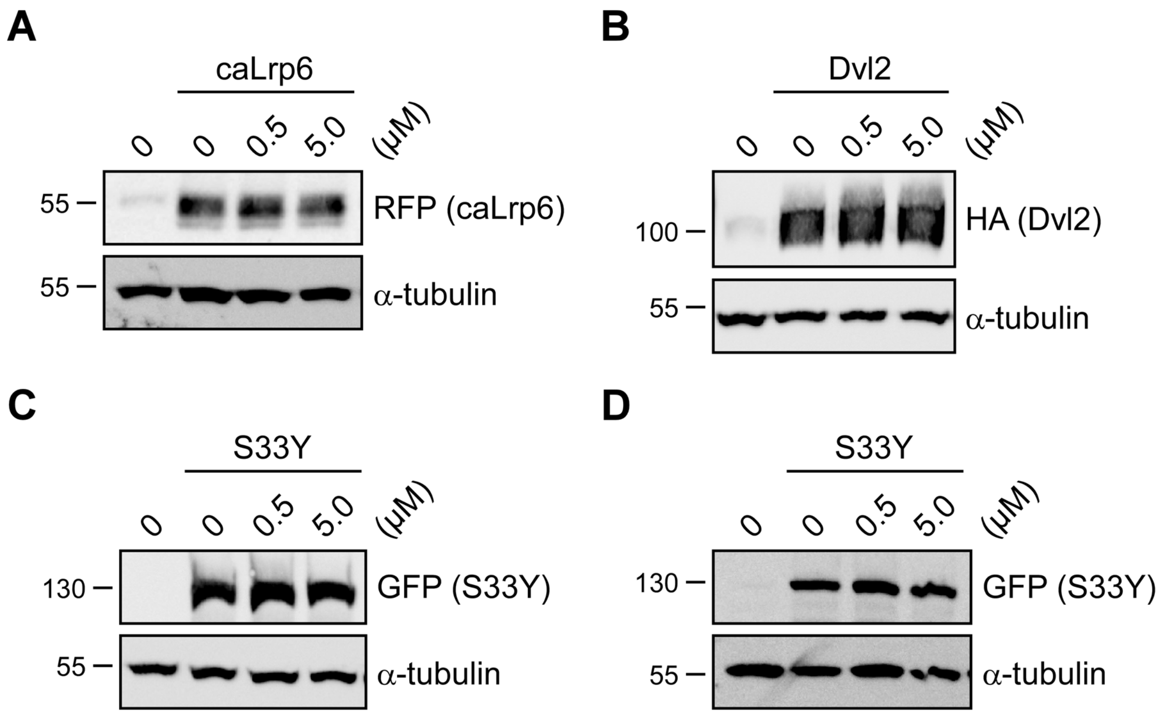

Supplementary Figure 5: SFN does not affect caLrp6, Dvl2 and S33Y levels. **(A-D)** Western blotting for RFP, HA and GFP in lysates of HEK293T **(A-C)** and DLD1 cells **(D)** which were used in the TOP/FOP luciferase reporter assays shown in Figure 5. A-D show the expression of caLrp6 to Figure 5C, of Dvl2 to Figure 5D, and of S33Y to Figure 5G and 5I, respectively. Loading control: α-tubulin.
